# Experimental study on collapsible and structural characteristics of artificially prepared loess material

**DOI:** 10.1038/s41598-023-31397-y

**Published:** 2023-03-13

**Authors:** Yuwei Zhang, Zhanping Song, Haochun Chen, Lei Ruan, Shimei He

**Affiliations:** 1 School of Civil Engineering, Xi’an University of Architecture and Technology, Xi’an, 710055 People’s Republic of China; 2 Key Laboratory of Geotechnical and Underground Space Engineering, Shaanxi Province/Xi’an University of Architecture and Technology, Xi’an, 710055 People’s Republic of China; 3 China State Construction Silkroad Construction Investment Group Co., Ltd., Xi’an, 710075 People’s Republic of China; 4 The 5th Engineering Co. Ltd. of China Railway Construction Bridge Engineering Bureau Group, Chengdu, 610500 China

**Keywords:** Structural geology, Civil engineering

## Abstract

Collapsibility and structural are two of the typical characteristics of natural undisturbed loess. It is of great significance to effectively simulate the collapsibility and structural of natural loess by preparing artificially loess. However, the existing methods of artificially preparing collapsible loess are complex, and the collapsibility of the prepared samples is difficult to control. In this paper, the collapsibility mechanism of loess was re-analyzed, and on this basis, a new method for preparing artificial collapsible loess using remolded loess, industrial salt, CaO particles and gypsum powder was proposed. The basic principle is: the CaO particles have structural strength and would transfer to Ca(OH)_2_ after soaking, this progress can simulate the disappearance of loess structural strength; The dissolution of industrial salt can simulate the collapse of loess internal pores, the collapsibility of artificial loess can be adjusted by adjusting the percentage of industrial salt; the gypsum powder can simulate the cementation of loess as a bonding material. The shear test, consolidation test and collapsibility test of artificially prepared loess and undisturbed loess were carried out. The test results of artificial loess were compared with undisturbed loess. The results show that: the plastic limit and liquid limit of the artificially prepared loess is smaller than that of the undisturbed loess; The optimal moisture content and maximum dry density are close to that of the undisturbed loess; The collapsibility coefficient of artificial prepared samples increases first and then decreases with the increase of load level, and gradually increases with the increase of industrial salt particle content; The structural parameters of artificially prepared loess samples first increase and then decrease with the shear process, but the structural parameters of artificial prepared loess and undisturbed loess are different under different confining pressure conditions.

## Introduction

Loess is widely distributed all over the world, such as America, Europe, Russia and China. Among them, China has the widest distribution area and the largest thickness^1^. As a typically structured soil, loess is characterized as porosity, structural and collapsible^2–8^. Accordingly, it is important to study collapsibility of loess for foundation design, as differential collapse deformations in loess could cause cracks in upper structures^9–13^. As is known to us all, physical model test is one of the proved method to investigate the influence of loess collapsibility on upper structure, however, the physical model test need sufficient undisturbed collapsible loess as dielectric material to conducted. During the sample collection, the porosity and structure is very easily to be disturbed. Moreover, the volume of the loess sample used in the model is large, impurities such as gravel and plant roots render natural structured soils in homogeneous and affect the soil structural characteristic. Therefore, preparing the artificial loess for the use of physical model test is an efficient method.

Up to now, some scholars have explored the feasibility and effectiveness of preparing artificial soils. The investigations suggests that the artificial soil is consistent with the natural undisturbed soil in homogenous structure and strength. The artificial loess is more suitable for the physical model test than the undisturbed loess. Maccarini (University of London, UK) first prepared artificial sands using combustion method. Thereafter, in order to prepare different kinds of artificial soils, different additives were added to the soil material by adjusting the mix. The additives include cement^14^, mixture of cement and ice particles^15^, copper slag^16^, gypsum^17^, industrial salt^18^, and other materials^19^. The artificial soil was an approval method to simulate the characteristic of natural undisturbed soil.

As to the artificial loess, Hu et al. prepared the artificial loess by pushing the CO_2_ to the mixture of CaO and the original loess, and compared its collapsibility with the undisturbed loess^20^. Zhang et al. made the strongly collapsible loess using the non-cohesive quartz powder, sand and adhesive bentonite, gypsum, industrial salt by the free fall method^21^. Assalla created the artificial loess by various methods and studied the collapse behaviour by oedometer experiments, the artificial samples mimic the bahaviour real loess remarkably well^22^; Jiang mixed CaO with original soil, compacted the mixture in layers to make samples, vacuum saturated the samples and then injected CO_2_ or wrapped the samples with enough Drikold to form CaCO_3_ cementation between particles^23^; Medero presented a collapsible soil was produced by adding particles of expanded polystyrene to a soil–cement mixture, collapse potential was evaluated on samples with and without cementation^24^; Arroyo prepared five different mixtures of soil (a granite saprolite) and cement (with cement contents in the range 0% to 7% on a dry weight basis), and tested its compression at four different water content levels^25^. As can be seen from above, the methods used to prepare the artificial loess is complicated and the collapsibility is difficult to be adjusted accurately.

In this paper, the collapsibility principle of loess was discussed from the perspective of microstructure. A new method of preparing artificial loess was presented. The disturbed loess, industrial salt, CaO and gypsum were taken as the original materials, the CaO and the industrial salt could simulate the soluble granule, the gypsum could simulate the cementing material. The structural strength and collapsibility strength of artificially prepared loess could be adjusted by changing the industrial salt. The basic physical parameters, shear strength parameters and structural parameters of artificial loess in different mixture design were tested. The best mixture design was suggested by comparing the artificial loess with the undisturbed loess. The artificial loess presented in this paper provide basis for the indoor physical model test.

## Preparation of artificial loess

### Principle of loess collapsibility

**Figure 1:**
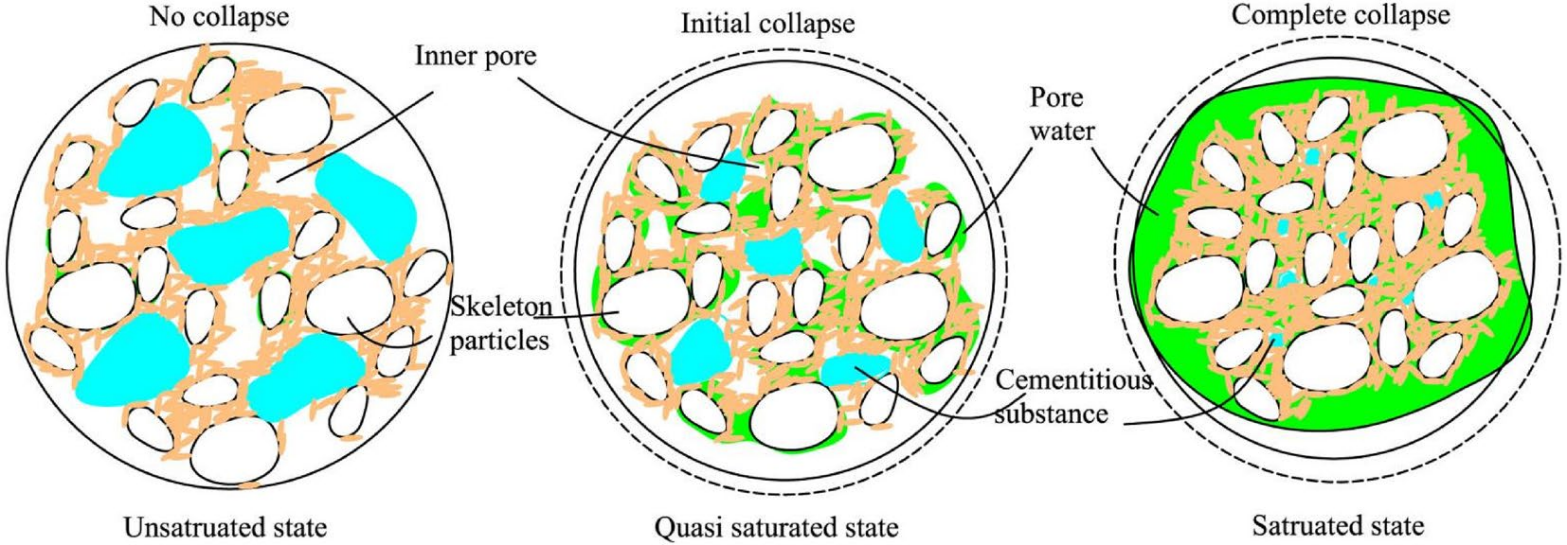
Collapsibility principle of natural loess form the perspective of microstructure.

The collapsibility is one of the typical characteristic of loess. Up to now, multiple theories about the principle of loess collapsibility have been presented. Among them, the most accepted theory by researchers is the theory of cementitious dissolution between particles^26^. According to the theory of cementitious dissolution, loess is composed of skeleton particles, cementitious substance and inner pores. Skeleton particles are connected with each other through cementitious substance, and certain pores are formed between skeleton particles. When the soil is in unsaturated state, the content of pore water in the pores is less, the cementitious substance has high bond strength, and the loess has obvious structure; When the water immersion reaches the quasi saturated state, the cementitious substance between the skeleton particles begins to dissolve, and the bonding strength decreases. Under the action of self weight load or external load, the macropores between the skeleton particles begin to collapse, and the relative displacement between the skeleton particles occurs, resulting in a certain amount of collapsing deformation; When the soil is completely saturated, the strength of cementitious substance between skeleton particles is completely invalid, and the macropores between skeleton particles are completely collapsed, the soil has obvious collapsing deformation. The collapsing progress is shown in Fig. 1. As can be seen from above, the cementation between the skeleton particles provides the bonding strength, which is the key to the formation of macropores in the loess. Due to the gradual increase of water content in the soaking saturation process, the bonding strength between the particles provided by cementation gradually disappears, which is the key to the collapse deformation of the loess. Therefore, in the preparation of artificial collapsible loess, using a suitable material to simulate the bond strength of cementitious material is the key to successful preparation.

### Preparation of samples

The previous researches show that the methods of preparing artificial loess is complex, and the collapsibility is difficult to control. It is of great significance to propose a new and effective preparation method of artificial collapsible loess. According to the collapse principle mentioned above, the fundamental reason for loess collapsing is that the cementation between particles dissolves in water and induces internal pore collapse. Considering the condition that CaO generates Ca(OH)_2_ when encountering water, which is partially soluble in water, so we used the CaO to simulate the structure of undisturbed loess. The process of CaO forming Ca(OH)_2_ and dissolving in water can be approximately regarded as the process of structural damage of undisturbed loess; Industrial salt can be completely dissolved in water, and the complete dissolution of industrial salt after loess is saturated with water can simulate the collapse process of pore structure in loess; Gypsum powder has high bonding strength under the condition of low water content, and gypsum powder gradually fails after being saturated with water. Therefore, gypsum powder is suitable to be used as a cementitious material of loess. So, in this paper, artificial collapsible loess is prepared by adding CaO particles, industrial salt and gypsum powder with remolded loess as the basic raw material. The collapsibility grade and structural strength of artificial loess could be changed by adjusting the percentage of CaO and industrial salt. The preparation method of artificial loess proposed in this paper closely fits the collapsible mechanism of loess, which is simpler and more effective than traditional methods. The method proposed in this paper provides a way for the large-scale preparation and application of artificial collapsible loess.

**Figure 2:**
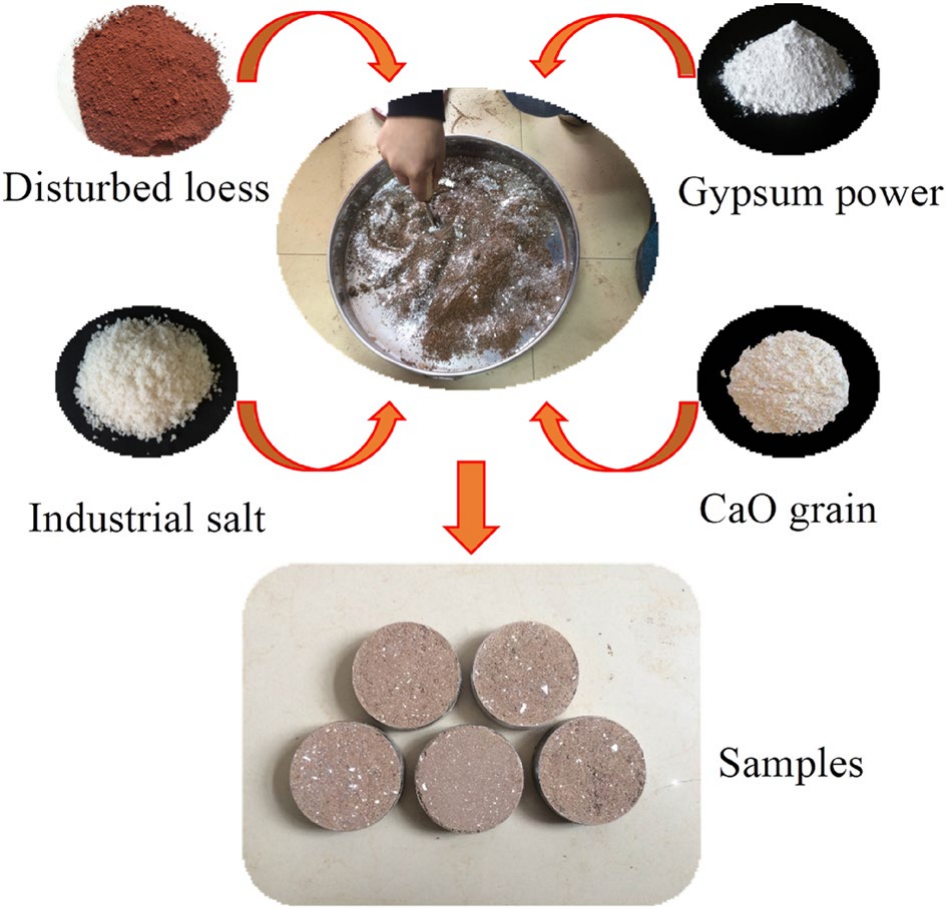
Testing materials.

When the artificial loess is used as the material for indoor model test, the amount is large, so the raw material for preparing artificial collapsible loess should be economical. The remolded loess, CaO particles and industrial salt selected in this paper have low cost, and the cost of gypsum powder is slightly higher, but the amount is less, so the overall cost is within a controllable range. The remolded loess is obtained by fully disturbing the natural undisturbed loess which was taken from Qujiangchi station of Metro line#4 in Xi’an. The physical parameters of undisturbed loess are shown in Table 1. The undisturbed loess was crushed into power and dry it, then pass it through a 2 mm sieve to obtain the remolded loess. The CaO was selected as particles with a diameter of about 1 mm. Industrial salt was also selected granular with a diameter of 0.5–1 mm; Gypsum powder adopts high-quality powdered gypsum powder. The testing materials are shown in Fig. 2.

**Table 1:** Parameters of undisturbed loess.

${G}_{s}$	Density/g/cm^3^	Moisture content/%	c/kPa	Void ratio
2.72	1.52	14.1	30.66	1.04

Compressibility modulus/MPa	Plastic limit	Liquid limit	Plastic limit index	
12.6	16.2	27.3	11.1

In the previous studies, the gypsum powder is mainly used as cementitious material to simulate the internal cementitious substance of loess. The results show that the simulation effect is good when the gypsum powder is 5% and 8%^21^. In this paper, the proportion of gypsum powder was also taken as 5% and 8%. The structural characteristic of undisturbed loess is controlled by the CaO grains, the proportion of CaO was taken as 1%. The collapsible characteristic of undisturbed loess was related to the proportion of industrial salt, the percentage of 2%, 4%, 6% and 8% was selected respectively to carry out the comparative tests. The specific proportion of each material is shown in Table 2. The shear tests, consolidation tests and collapsibility tests were conducted respectively used the artificial prepared loess and natural undisturbed loess.

**Table 2:** Material proportion of artificially prepared samples.

Sample number	Remolded loess/%	Industrial salt/%	Gypsum powder/%	CaO/%
Sample1	92	2	5	1
Sample2	90	4	5	1
Sample3	85	6	8	1
Sample4	83	8	8	1

In order to better compare the collapsibility and structure of artificially prepared loess and undisturbed loess, the density and moisture content of the artificially prepared soil sample are consistent with the undisturbed loess. The artificial soil samples can be obtained by pressing sample method. The specific steps pressing sample method are as follows: firstly, the total mass of the remolded loess, CaO particles, industrial salt and gypsum powder was calculated from the volume of the test sample (such as the large ring knife of the collapsible test, the small ring knife of the shear test, etc.); secondly, the mass of each component required for each sample could be calculated according to the sample ratio table; Thirdly, the water consumption can be calculated by combining the moisture content. In this paper, the large ring knife sample was taken as an example to achieve the mass of each component of the sample using the above method, the results are shown in Table 3. After obtaining the mass of each component, the required ring knife sample can be directly compacted through the sample pressing method (Fig. 2). In this way, we can achieve the artificial soil samples, which is similar to the undisturbed soil. The required test samples are prepared according to the above methods. It should be noted that only remolded soil is configured during the water content configuration in the sample preparation process and the water will be fully and evenly blocked for 48 h. Then the CaO particles, industrial salt and gypsum powder was mixed with the remolded soil. The CaO particles cannot be pre mixed with water, otherwise there will be a reaction and its structure cannot be simulated.

**Table 3:** Material quality of big ring knife samples.

Sample number	Remolded loess/g	Industrial salt/g	CaO/g	Gypsum powder/g	Water/g
Sample1	122.76	2.67	1.33	6.67	18.82
Sample2	120.01	5.34	1.33	6.67	18.82
Sample3	113.42	8.01	1.33	10.68	18.82
Sample4	110.76	10.68	1.33	10.68	18.82

## Testing methods

It is difficult to make all the mechanical parameters of artificially prepared loess completely consistent with the undisturbed loess. In this paper, the purpose of preparing collapsible loess is to be used as simulation material for indoor model test, the main concern is whether the basic mechanical parameters, collapsibility and structural parameters of artificially prepared loess are consistent with those of undisturbed loess. Therefore, the basic physical parameters of artificial loess are tested firstly, then the strength parameters, deformation parameters, collapsibility coefficient and the structural parameter were tested by shear test, consolidation test, collapsibility coefficient test and triaxial shear test.

### Direct shear test

**Figure 3:**
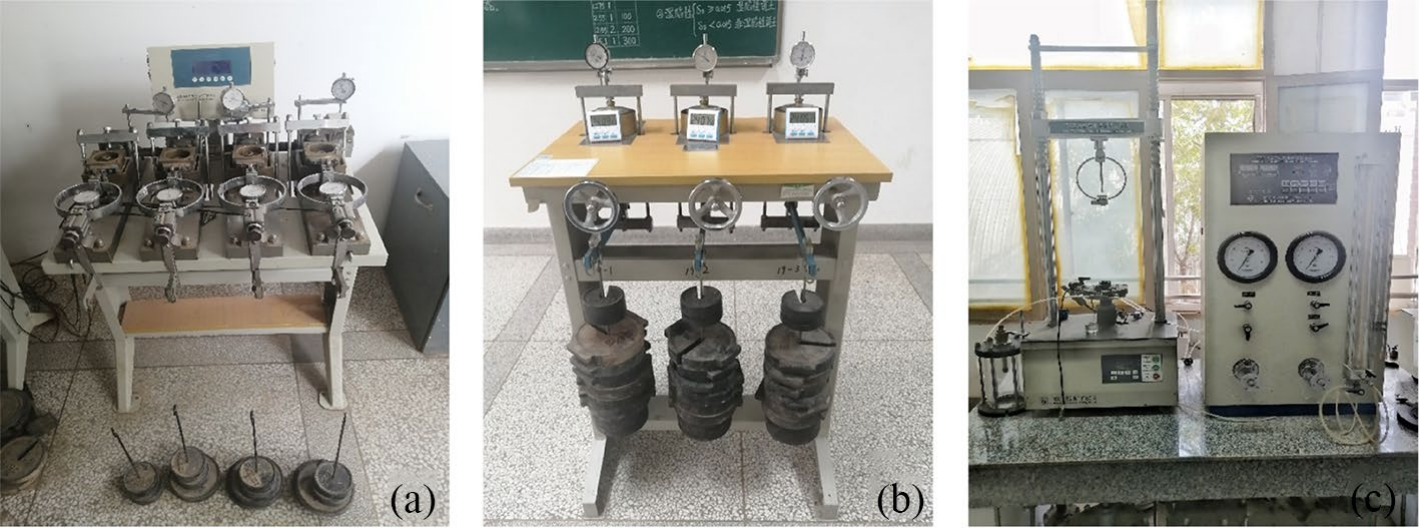
Testing equipment.

The strength parameters of loess could be tested by the direct shear test. The ZJ type quadruple strain controlled direct shear apparatus produced by Nanjing Soil instrument company was used in the direct shear test (shown in Fig. 3a). The strain type direct shear instrument is composed of a shear box, a vertical loading device, a shear transmission device, a force measuring ring, and a displacement measurement system (the division value of the gauge is 0.01 mm, the measuring range is 10 mm, and the accuracy of the sensor is zero). The vertical pressure of the test piece is 50 kPa, 100 kPa, 200 kPa and 300 kPa respectively. The test process was carried out in strict accordance with the requirements of <highway geotechnical test code 2007>.

### Consolidation test

The deformation parameters of loess could be tested by the consolidation test. The WG single lever consolidator was used for consolidation test (shown in Fig. 3b). The consolidation pressure of the consolidation test is 50 kPa, 100 kPa, 200 kPa, 300 kPa and 400 kPa respectively, and the next level of load can be loaded only after the deformation of the previous level of load is stable (the deformation within 1 h is less than 0.01 mm).

### Collapsibility test

The collapsible coefficient can be tested by the collapsibility test. The WG single lever consolidator was also used for collapsibility test. The collapsibility test was carried out by the single line method, and the vertical stress of five samples was also set to 50 kPa, 100 kPa, 200 kPa, 300 kPa and 400 kPa. According to the test requirements, each sample was also loaded in stages (50 kPa per stage), and the next stage of loading was carried out after the load and deformation of each stage reached stability. The sample can be soaked until the deformation of the last stage reached stability. The collapsibility coefficient under different self weight pressures of each sample would be obtained.

### Triaxial shear test

The structural characteristic is one of the typical characteristic of loess. Xie et al. proposed the expression of structural parameter: ${m}_{\sigma }={\left({\sigma }_{1}-{\sigma }_{3}\right)}_{o}^{2}/\left[{\left({\sigma }_{1}-{\sigma }_{3}\right)}_{r}{\left({\sigma }_{1}-{\sigma }_{3}\right)}_{s}\right]$, where ${m}_{\sigma }$ is the stress type structural parameters, ${\left({\sigma }_{1}-{\sigma }_{3}\right)}_{o}$, ${\left({\sigma }_{1}-{\sigma }_{3}\right)}_{r}$, ${\left({\sigma }_{1}-{\sigma }_{3}\right)}_{s}$ are the corresponding shear stress values of undisturbed loess, remolded loess and saturated loess at the shear strain $\varepsilon$. In order to study the differences of structural characteristic between artificial loess and undisturbed loess, the triaxial tests of undisturbed, remolded and saturated soil samples were also carried out. The triaxial tests were conducted using the triaxial shear apparatus shown in Fig. 3c.

## Results and discussion

### Strength parameters

Firstly, the basic physical parameters of each sample of artificially prepared samples were measured. The liquid limit moisture content and plastic limit moisture content of each sample were measured by the liquid plastic limit joint determination method. The moisture content corresponding to the 76 g cone with the soil depth of 2 mm was taken as the plastic limit, and the moisture content corresponding to the soil depth of 17 mm was taken as the liquid limit. The best moisture content and maximum dry density of each sample were obtained by standard compaction test. The basic physical parameters of each sample were finally shown in Table 4.

**Table 4:** Basic physical parameters of artificial samples.

Sample number	Liquid limit/%	Plastic limit/%	Plasticity index	Optimum moisture content/%	Maximum dry density/(g/cm^3^)
Sample1	26.6	15.8	10.8	16.1	1.75
Sample2	26.3	16.2	10.1	15.8	1.67
Sample3	25.8	16.3	9.5	16.2	1.73
Sample4	25.3	16.4	8.9	15.4	1.63

**Figure 4:**
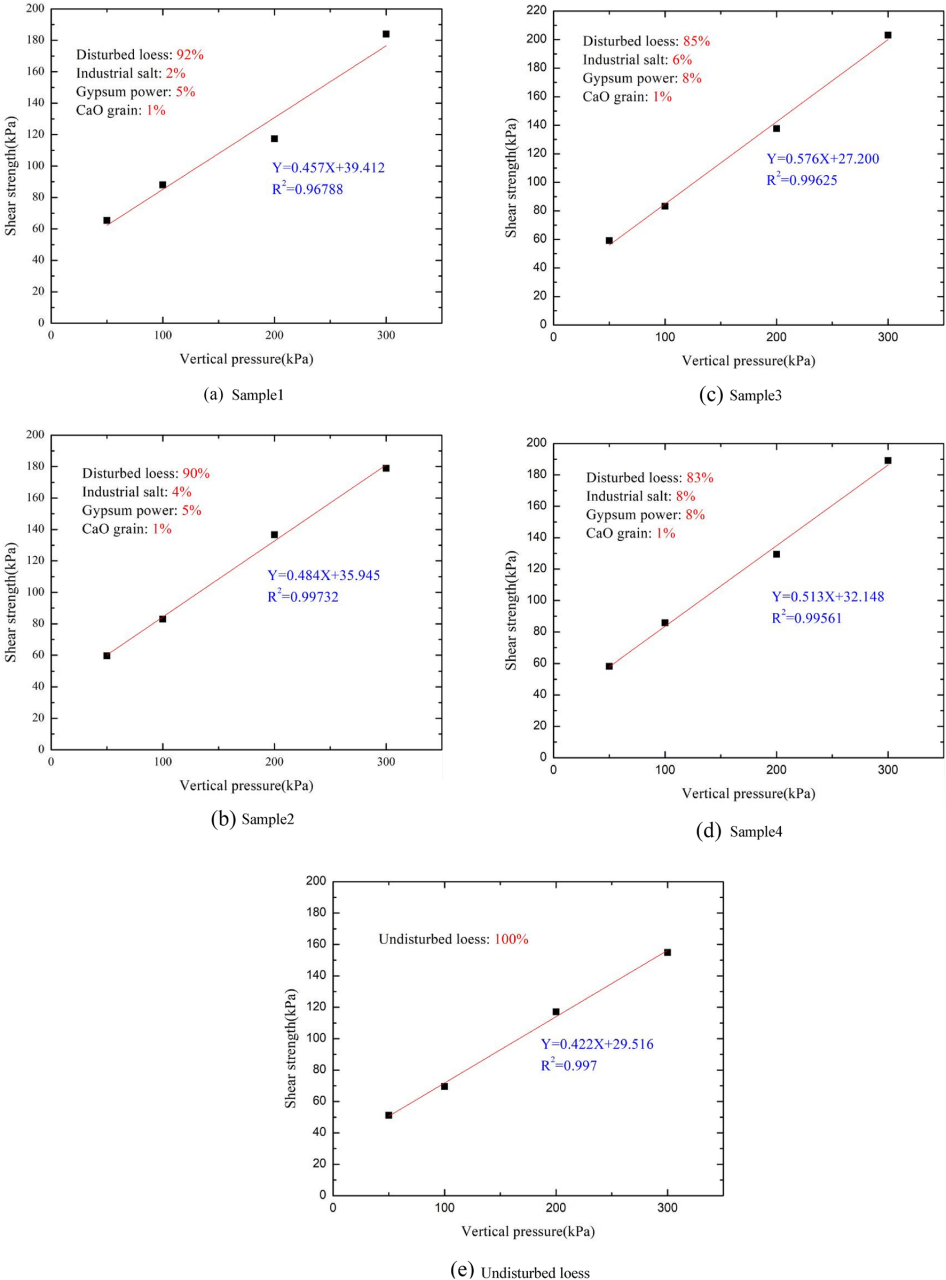
Results of direct sheer tests.

In order to analyse the shear strength characteristics, the direct shear tests of undisturbed loess samples and artificially prepared samples were carried out respectively. Small ring knife samples with a diameter of 61.8 mm were selected, the shear speed was 0.8 mm/min, and the vertical pressure was taken as 50 kPa, 100 kPa, 200 kPa and 300 kPa respectively. The relationship between the shear strength and the vertical pressure of each sample was obtained (see in Fig. 4). The values of $\mathrm{cohesion }c$, internal friction angle $\varphi$ of different artificially prepared samples were obtained by curve fitting, as shown in Table 5.

**Table 5:** Parameters of shear strength.

Sample number	$c$/kPa	$\varphi$/°
Sample1	39.48	24.6
Sample2	35.1	26.2
Sample3	31.67	27.4
Sample4	28.12	29.7
Undisturbed loess	30.66	22.7

As can be seen from Fig. 4 and Table 5, the cohesion $c$ and the internal friction angle $\varphi$ are related to the content of industrial salt and gypsum powder. With the increase of industrial salt content, the cohesion gradually decreases and the internal friction angle gradually increases. With the increase of gypsum power, internal friction angle gradually increases the cohesion of sample 3 is close to that of undisturbed loess, but the internal friction angle is relatively smaller than that of undisturbed loess. In view of the internal friction angle is related to gypsum powder content, it can be considered to change the internal friction angle by changing the gypsum powder content.

### Deformation parameters

**Figure 5:**
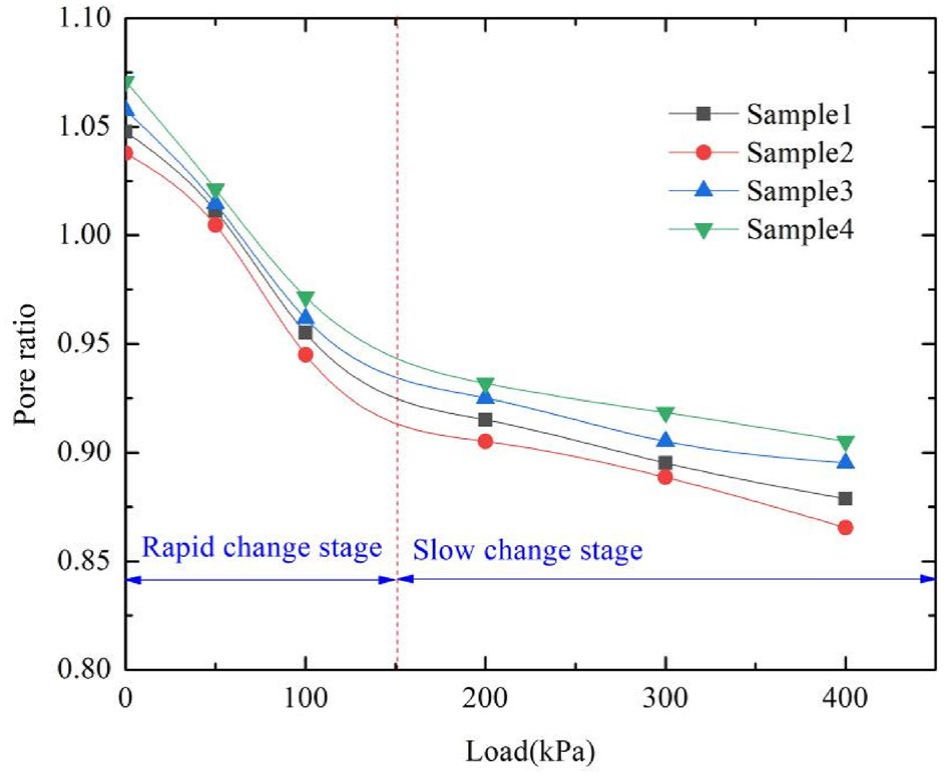
Results of consolidation test.

The standard consolidation tests of artificial prepared samples were carried out. The consolidation load levels are selected as 50 kPa, 100 kPa, 200 kPa, 300 kPa and 400 kPa respectively. The $e-p$ curve was drawn in Fig. 5. The compressibility coefficient and compressibility modulus were calculated according to the specification formula, and the results are shown in Table 6.

**Table 6:** Parameters of consolidation test.

Sample number	Compressibility coefficient/MPa^−1^	Compressibility modulus/MPa
Sample1	0.41	4.76
Sample2	0.39	4.98
Sample3	0.36	5.45
Sample4	0.31	5.88
Undisturbed loess	0.34	5.77

As can be seen from Fig. 5, with the increase of load, the void ratio of the sample decreases gradually. The compression process can be divided into two stages: rapid change stage and slow change stage. The mix proportion has influence on the compressibility of samples. As can be seen from Table 6, the compressibility coefficient and compressibility modulus are related to the content of industrial salt and gypsum powder. With the increase of industrial salt content, the compression coefficient decreases and the compression modulus increases. With the increase of gypsum powder, the compression modulus increases. The compressibility coefficient and compressibility modulus of sample 3 are closest to undisturbed loess.

### Collapsibility coefficient

**Figure 6:**
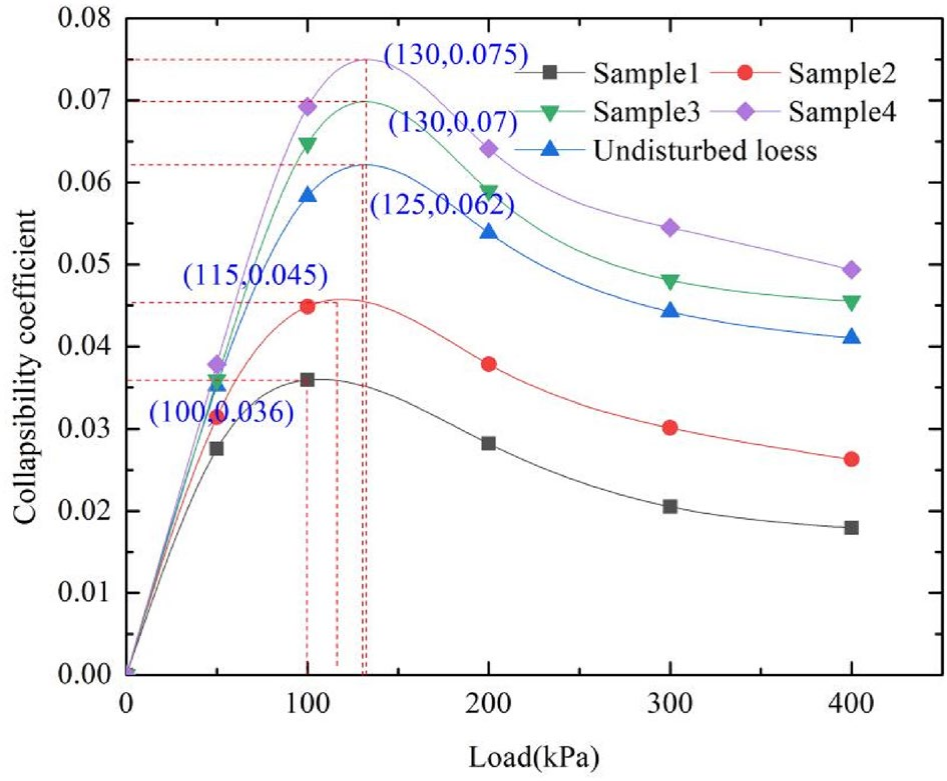
Collapsibility coefficient of different load.

The collapsibility coefficient is one of the important parameters of loess. Collapsibility is the key index for the success of artificial loess preparation. In this paper, we tested the collapsibility of artificial samples and undisturbed loess using the single line method. In order to measure the collapsibility coefficient under different pressures, the load levels are selected as 50 kPa, 100 kPa, 200 kPa, 300 kPa and 400 kPa respectively. The collapsibility coefficients of each sample under different load levels are shown in Fig. 6.

As can be seen from Fig. 6, the collapsibility coefficient increases first and then decreases with the increase of external load. This is because both artificially prepared loess and undisturbed loess have inherent structural strength. The collapsibility coefficient of sample 1 is smallest, the external load required to reach the maximum collapsibility is 100 kPa; The collapsibility coefficient of sample 4 is biggest, the external load required to reach the maximum collapsibility is 130 kPa. When the external load is less than the structural strength of the loess, the soil consolidation is insufficient, and the collapsibility coefficient will increase with the increase of external load. If the external load is greater than the structural strength of the loess, the soil will have preconsolidation effect, and the internal pores will be compressed. At this time, the collapsibility coefficient will decrease. The collapsibility coefficient of artificial prepared loess is obviously affected by the coupling effect of load and soaking due to the existence of inherent structural strength. Under the same load level, the collapsibility coefficient of artificial prepared samples increases with the increase of industrial salt content, however, the increase effect is not obvious at the low load level. The collapsibility coefficient of sample 3 is close to that of undisturbed loess. Take sample 3 as an example to analyze the cumulative collapsibility of samples under different load levels.

**Figure 7:**
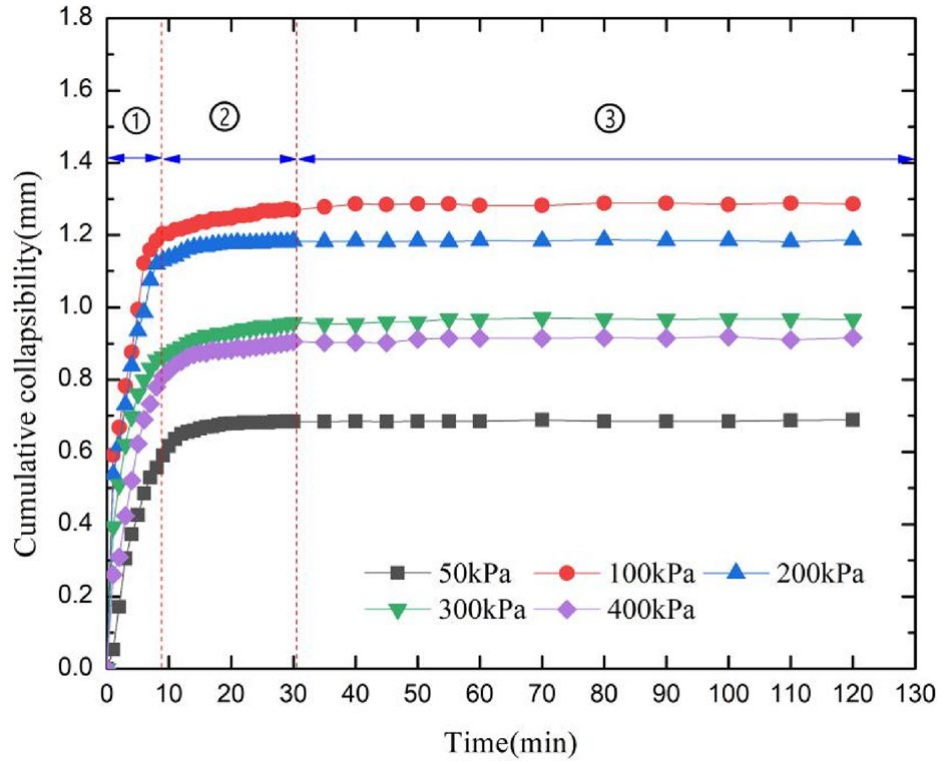
Cumulative collapsibility of sample 3.

The cumulative collapsible deformation of sample 3 under different load levels was shown in Fig. 7. As can be seen from Fig. 7, The change curve of cumulative collapsibility with time can be divided into three stages: ① the rapid change stage, ② the slow change stage, ③ the stable stage. The smaller the load level is, the slower the initial deformation rate of the sample is, and the smaller the final cumulative collapsible deformation is. With the increase of load level, the initial deformation rate and cumulative collapsible deformation increase. When the external load is 100 kPa, the final cumulative collapsible deformation is the largest. When the load is further increased, the final cumulative collapsible deformation of the sample decreases due to pre consolidation effect. This result indicating that when the external load level is close to the structural strength of the sample, its collapsibility is the most obvious. Therefore, when evaluating the collapsibility of loess in practice, the upper load and preconsolidation of loess should be fully considered.

### Structural parameters

According to the above analysis, the strength parameters, deformation parameters and the collapsibility coefficient of sample 3 are closest to that of undisturbed loess. We selected the sample 3 to analyse the structural parameters. According to the expression of structural parameter proposed xie et al.: ${m}_{\sigma }={\left({\sigma }_{1}-{\sigma }_{3}\right)}_{o}^{2}/\left[{\left({\sigma }_{1}-{\sigma }_{3}\right)}_{r}{\left({\sigma }_{1}-{\sigma }_{3}\right)}_{s}\right]$, where ${m}_{\sigma }$ is the stress type structural parameters, ${\left({\sigma }_{1}-{\sigma }_{3}\right)}_{o}$, ${\left({\sigma }_{1}-{\sigma }_{3}\right)}_{r}$, ${\left({\sigma }_{1}-{\sigma }_{3}\right)}_{s}$ are the corresponding shear stress values of undisturbed loess, remolded loess and saturated loess at the shear strain $\varepsilon$. Triaxial shear tests of artificial sample 3, undisturbed loess, remodeled loess and saturated loess were conducted. The structural parameters of undisturbed loess and artificial sample 3 were calculated by the method above.

**Figure 8:**
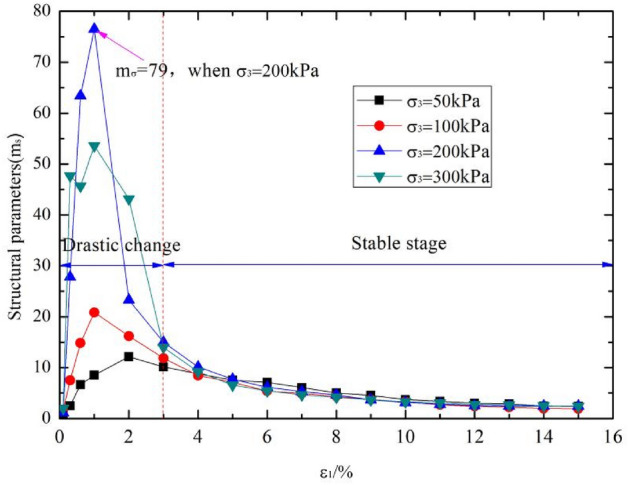
Variation law of structural parameters of sample 3.

**Figure 9:**
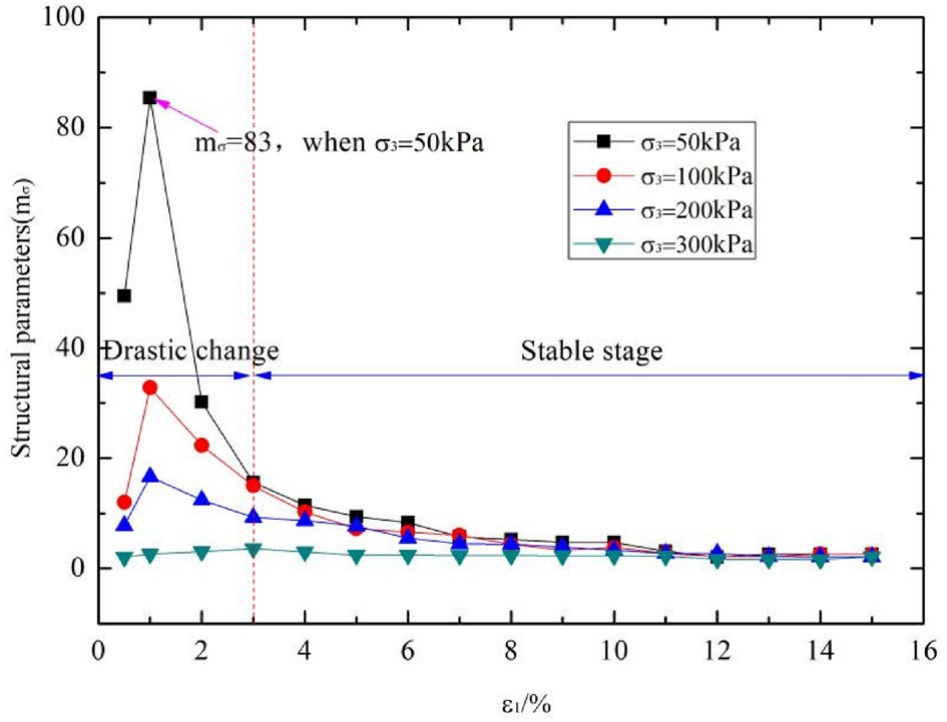
Variation law of structural parameters of undisturbed loess.

The evolution law of structural parameters of sample 3 and undisturbed loess with triaxial shear process are shown in Figs. 8 and 9 respectively. It can be seen that the structural parameters first increases and then gradually tends to be stable with the shear failure of loess (the shear strain value increases) under different confining pressures. This indicate that the potential structural is gradually released during the shear process. After the shear failure, the structure of loess gradually tends to disappear, and the structural parameters gradually tend to zero. It also can be seen that the changes of structural parameters were different under different confining pressures. When the confining pressure is 200 kPa, the structure of artificial loess is the most obvious. This is due to the fact that when the confining pressure is small, the binding effect of external confining pressure on soil is not obvious, and the performance of structural strength is not significant. With the increase of confining pressure, the binding effect of external action on loess gradually increases, which is equivalent to increasing the shear strength of soil and resulting in the increase of structural strength of soil. However, when the confining pressure further increases, the structure will decrease, which may be due to the external confining pressure exceeding the structural strength of the soil, causing disturbance to the soil, which is equivalent to deformation destroying of the structural strength of the soil and resulting in the reduction of its structural parameters. When the confining pressure is 50 kPa, the structure of undisturbed loess is the most obvious. With the increase of confining pressure, the structural parameters gradually decrease. From the overall results, the structural evolution law of artificially prepared collapsible loess is consistent with that of undisturbed loess.

### Microstructure

**Figure 10:**
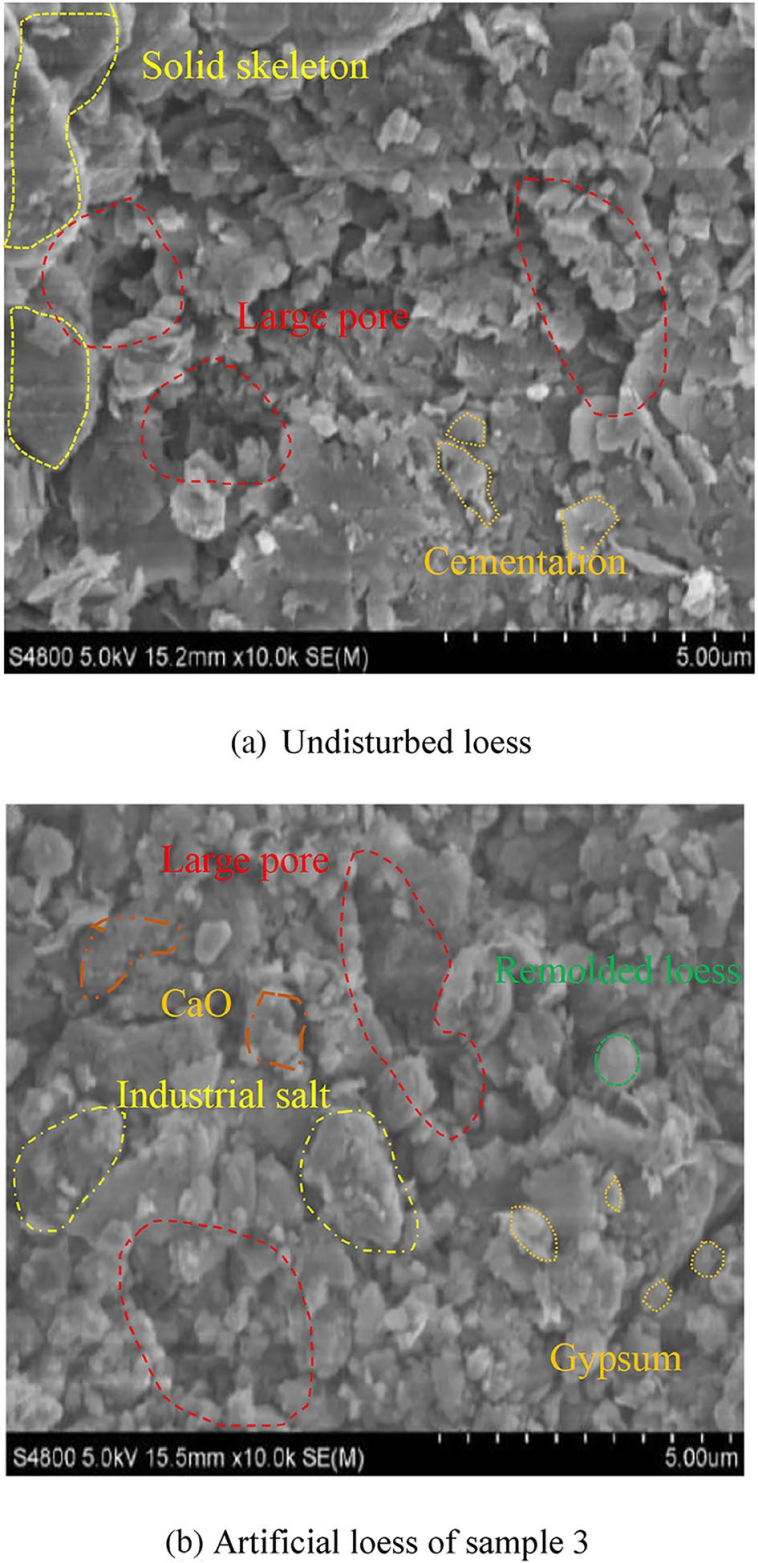
Microstructure images.

In order to observe the internal pore structure of artificial loess and undisturbed loess, some SEM tests were conducted by the S-4800 cold field emission scanning electron microscope. The results were shown in Fig. 10. As can be seen from Fig. 10a, the solid skeleton is obvious. Solid particles are connected by cementitious substance to form the solid skeleton. The large pore distribute in the solid skeleton. The cementation provide the bond strength with low water content. With the increase of water content, the bond strength of cementation will disappear gradually, and the large pore will collapse. As for Fig. 10b, there are remolded loess particles, industrial salt, CaO particles distributed in the internal soil. Some large pore exist in the soil but not so obvious compared with the undisturbed loess. The industrial salt particles is obvious, the industrial salt will dissolve in water gradually with the increase of water content. This process could simulate the collapse of undisturbed loess. Therefore, the artificial loess have the same collapse characteristic with the undisturbed loess form the microstructure results.

## Conclusions

In this paper, the collapsible principle of natural undisturbed loess was discussed. Based on the collapsible principle a new method of preparing artificial loess using remolded loess, CaO particle, industrial salt and gypsum powder was proposed. The direct shear test, consolidation test, collapsibility coefficient test and triaxial shear test were carried out to investigate the main characteristic of artificial prepared loess and undisturbed loess. The main remarks are as follows:

1. The shear test and consolidation test show that with the increase of the content of industrial salt, the cohesion of the artificially prepared collapsible loess gradually decreases, the internal friction angle gradually increases, the compression coefficient decreases, and the compression modulus increases.
2. Both material ratio and load level have an impact on the collapsibility of artificially prepared loess. Under the same material ratio, the collapsibility coefficient increases first and then decreases with the increase of external load. Under the same load level, the collapsibility coefficient of artificially prepared samples increases with the increase of industrial salt particle content.
3. The variation law of structural parameters of artificially prepared loess samples is similar to that of undisturbed loess. The structural parameters first increase and then decrease with the shear process. However, due to the limitations of artificial prepared samples, the structural parameters of artificially prepared loess under different confining pressure conditions are different.

## Data Availability

Data will be made available on request. Yuwei Zhang should be contacted if someone wants to request the data from this study.
